# State Board of Health

**Published:** 1876-05

**Authors:** 


*State Board of Health.*—We had supposed the law organizing a Board of Health in the State of Georgia had been generally distributed throughout the State by the Secretary, but find that it is very imperfectly understood. We will, therefore, for the information of our readers, publish this law in full in our next issue, as originally enacted, with the amendments adopted by the last Legislature.

☞ Always read our advertisements and present their claims to your home druggist. Advise your friends to send for I. S. McCreary circular, and to subscribe for Appleton's American Cyclopædia.

